# Morphology Control of TiO_2_ Nanorods Using KBr Salt for Enhancing the Photocatalytic Activity of TiO_2_ and MoS_2_/TiO_2_ Heterostructures

**DOI:** 10.3390/nano12172904

**Published:** 2022-08-24

**Authors:** Zeineb A. Thiehmed, Talal M. Altahtamouni

**Affiliations:** Materials Science & Technology Program, College of Arts & Sciences, Qatar University, Doha P.O. Box 2713, Qatar

**Keywords:** hydrothermal, morphology control, TiO_2_ nanorods, photocatalysis, heterostructure

## Abstract

In this study, the effect of KBr salt on the growth of TiO_2_ nanorods (NRs) was systematically studied. The addition of KBr with different concentrations provides a controllable growth of TiO_2_ NRs using hydrothermal method. The results revealed that the presence of KBr molecules affects the growth rate by suppressing the growth in the lateral direction and allowing for axial growth. This results in affecting the morphology by decreasing the diameter of the nanorods, and increasing the free space between them. Enhancing the free spaces between the adjacent nanorods gives rise to remarkable increase in the internal surface area, with more exposure side surface. To obtain benefit from the enlargement in the inner surface area, TiO_2_ NRs were used for the preparation of MoS_2_/TiO_2_ heterostructures. To study the influence of the morphology on their activity, TiO_2_ NRs samples with different KBr concentrations as well as the MoS_2_/TiO_2_ heterostructures were evaluated towards the photocatalytic degradation of Rhodamine B dyes.

## 1. Introduction

TiO_2_ nanostructures have attracted an extensive attention over the last years, owing to their unique properties for several applications such as catalysis, solar cells, and biochemical applications [1,2,3,4,5,6]. TiO_2_ is a famous wide-band semiconductor with high corrosion resistance, low cost, low toxicity, and good chemical stability. Moreover, due to their unusual electrical and optical activity, TiO_2_ nanostructured materials have been used in sensors, photocatalysis, perovskite solar cells, and batteries [7,8,9]. It is well known that the properties and the behavior of the TiO_2_ are essentially dependent on the microstructure of the material, such as size, orientation, and morphology. Therefore, achieving a controllable synthesis over TiO_2_ nanostructures is a significant target to tailor their behavior and enhance their activity. However, though among all the TiO_2_ crystal phases, anatase phase has been extensively investigated as the most active photocatalyst, excellent photocatalysis properties of rutile phase have also been disclosed [10,11,12,13]. This achieved photocatalytic activity of the rutile phase is attributed to the synthesis of nanostructures with enhanced surface area and more exposed active sites. Among all the TiO_2_ nanostructures, 1D TiO_2_ nanorods (NRs) exhibit significant photocatalytic activity enhancement, providing a direct pathway for the charge carriers and a good side exposure for reactions. This can provide more active sites, and increase the electron transport rate, leading to enhanced performance [14,15,16]. The properties of TiO_2_ NRs depend mainly on the preparative method and its respective parameters. Numerous synthetic techniques, such as sol-gel, hydrothermal, electron beam evaporation, and spray pyrolysis, have been utilized to construct TiO_2_ NRs [17,18,19,20]. Among them, hydrothermal technique is a powerful low-temperature method with inexpensive mass production of TiO_2_ NRs. This method allows a direct growth of well-aligned TiO_2_ NRs on the substrate surface. Fluorine-doped tin oxide (FTO) has been intensively used as a glass substrate for optical and electrical applications of TiO_2_ NRs, due to its high transparency, low resistivity, and good chemical/thermal stability. Moreover, FTO has a minor lattice mismatch and the same tetragonal crystal structure as TiO_2_. This can provide feasible nucleation and epitaxial growth of the nanorods [21,22,23]. However, there is still a struggle in obtaining the desired shape, orientation, and size of the grown nanorods. Several studies have been conducted to control the size of the grown nanocrystals by tuning the synthesis parameters such as time, temperature, reactant concentration, and titanium precursor. Another parameter that can affect the growth of the nanocrystals is adding surfactants or salts. Adding salts can provide significant control over the crystal size of the grown nanostructures. Few studies were conducted to investigate the effect of adding NaCl salt to the growth solution [15,24,25]. The results revealed that addition of NaCl molecules provides a control over the crystal growth of the nanostructures. In the case of NRs structure, this control may improve the photocatalytic activity of the NRs in two perspectives. The first point is decreasing the diameters of the NRs may provide more exposed side surfaces. This is expected to enlarge the inner surface area of the NRs, with more exposure to the light for photocatalytic applications. The second perspective is increasing the internal surface area of the NRs, so the larger space between the adjacent NRs would be utilized for the construction of heterostructures between the NRs and other suitable materials. Constructing the semiconductor heterostructures by coupling TiO_2_ with suitable materials has been shown to be an effective approach to enhance the photocatalytic performance. Heterostructure serves three main purposes, which are: (i) to enhance the visible light absorption, (ii) to improve the charge separation, and (iii) to increase the lifetime of charge carriers. Wan et al. reported the effect of etching treatment on TiO_2_ NRs, where enlarging the gap space between the NRs enhanced the loading of the CdS quantum dots, providing improved photovoltaic performance [26].

Up to our knowledge, NaCl is the only salt that has been examined for tuning the morphology of TiO_2_ nanocrystals. Hence, this work provides a controllable synthesis of TiO_2_ NRs via KBr salt addition to the growth solution. The effect of KBr on the morphology, crystallinity, and the photocatalytic activity towards dye degradation of the grown NRs was investigated. By tuning the size, orientation, and the internal surface area of the grown NRs, MoS_2_/TiO_2_ heterostructures were prepared via chemical vapor deposition technique. MoS_2_ is a promising member of TMDs family, where both theoretical and experimental studies pointed out its strong catalytic activity. Nanostructures of MoS_2_ exhibit a direct band gap with around 1.86 eV, which makes it a suitable candidate to form a type-II alignment heterostructure with TiO_2_. Thus, coupling it with TiO_2_ is believed to enhance the photocatalytic activity. This will also open the door for exploring other TMDs member such as WS_2_ and MoSe_2_ and WSe_2_. The prepared MoS_2_/TiO_2_ heterostructure was tested for photocatalytic dye degradation application towards RhB.

## 2. Experimental Section

### 2.1. Materials and Methods

#### 2.1.1. Materials

Titanium (IV) butoxide (C_16_H_36_O_4_Ti (97%, Sigma Aldrich, Burlington, MA, USA)), hydrochloric acid (HCl (37%, Analar NORMAPUR, Pennsylvania, PA, USA)), and potassium bromide (KBr (99.97%, Sigma Aldrich, Burlington, MA, USA)) were used as precursors for the hydrothermal synthesis. Molybdenum trioxide (MoO_3_, 99.97%, Sigma Aldrich, Burlington, MA, USA) and sulfur (S, 99.5%, Sigma Aldrich, Burlington, MA, USA) in a powder form were used as precursors for the synthesis of synthesis of the heterostructure using chemical vapor deposition technique. Before the growth, FTO substrates were cleaned in an ultrasonic bath using acetone, ethanol, and deionized water for 15 min each. After that, the substrates were dried using nitrogen flow.

#### 2.1.2. TiO_2_ NRs Synthesis Method

The fabrication of TiO_2_ NRs was conducted via hydrothermal method. First, freshly prepared 30 mL aqueous hydrochloric acid was obtained by mixing 15 mL concentrated HCl (37%) with 15 mL deionized water. After a gentle stirring for 10 min, the addition of 0.3 mL Titanium butoxide took place, followed by continuous stirring for 15 min. Meanwhile, the cleaned FTO substrates were arranged inside a 100 mL Teflon-lined stainless-steel autoclave, with the FTO conductive side facing the wall of the Teflon beaker. Subsequently, the prepared solution was poured into the Teflon beaker. The hydrothermal synthesis was conducted in electric oven at 160 °C for 10 h, and then cooled down to room temperature. The prepared TiO_2_ NRs on FTO substrates were extensively rinsed with deionized water and dried using nitrogen flow. To investigate the effect of adding KBr salt on the TiO_2_ NRs properties, different growth solutions with different KBr concentrations (0 M, 0.05 M, 0.1 M, and 0.15 M) were prepared.

#### 2.1.3. MoS_2_/TiO_2_ Heterostructure Synthesis Method

For the synthesis of MoS_2_/TiO_2_ heterostructures, a TiO_2_ NRs sample were utilized to act as a substrate for the CVD deposition of MoS_2_ nanoflakes on the TiO_2_ NRs. The growth took place in a Lindberg Blue M CVD system, using a 2-inch diameter quartz tube placed inside tubular furnace. The powder precursors used where molybdenum trioxide (MoO_3_, 99.97%, Sigma Aldrich, Burlington, MA, USA) as a source of Mo, and sulfur (S, 99.5%, Sigma Aldrich, Burlington, MA, USA). The used carrying gas was argon (Ar, 99.999%). In typical growth, 20 mg of MoO_3_ were weighted and placed in an alumina ceramic boat, then clean FTO substrate was placed on the middle top of that boat, with its surface facing down. Then this ceramic boat was placed at the center of the quartz tube. Two hundred milligrams of sulfur were weighted and kept in a ceramic crucible, which was placed upstream the tube. After evacuation of the reactor, the quartz tube was purged with 100 sccm Ar flow for 15 min. Then, the temperature of the furnace was gradually increased with a ramping rate of 15 °C/min until it reached 675 °C. The deposition took place at this temperature for 20 min under 100 sccm Ar flow. After 20 min growth, the furnace was turned off and cooled down to room temperature naturally, under 500 sccm Ar flow.

### 2.2. Characterization

To analyze the microstructure and the morphology of the samples, transmission electron microscope (Thermo Fisher Scientific TalosF200X TEM (Waltham, MA, USA) coupled with a SuperX EDS system (Waltham, MA, USA)) and scanning electron microscope (SEM, Nova Nano 450, Hillsboro, Ore, USA) were utilized. STEM micrographs were recorded with a High-angular Annular Dark Field Detector. TEM images were acquired using BM-CETA 16 M CCCD camera. Raman spectra of the samples were provided using Thermo fisher scientific (DXRTM 2 Smart Waltham, MA, USA) Raman Microscope with a wavelength of 532 nm, 40 times scanning, and the laser power used was 10 using 50× microscope objectives, at room temperature. The Raman signals were collected using a 900 lines/mm grating by a charge-coupled device (CCD) detector. PANalytical EMPYREAN Bragg–Brentano X-ray powder diffraction, 40 KV/30 mA, and Cu-Kα radiation (λ = 1.54056 Å) was used with a scan rate of 2°/min to study crystal structure of the samples.

### 2.3. Photocatalytic Degradation of Rhodamine B

The photocatalytic degradation activity of the synthesized TiO_2_ NRs as well as the prepared MoS_2_/TiO_2_ heterostructure were investigated against Rhodamine B dye by monitoring photo-assisted degradation. The experiments were performed under the radiation of 150 W Xe arc solar simulator with an AM 1.5 G filter as the light source. Each sample was immersed in 50 mL Rhodamine B dye (10 ppm) with continuous stirring. Prior to the radiation exposure, the sample was kept in dark for 30 min. Later on, the solution was exposed to solar simulation radiation with a distance of 20 cm above the solution surface. At a fixed interval of 30 min for 2 h, 3 mL of the dye solution was collected to examine its absorption spectra at characteristic peak located at 554 nm. The photocatalytic activity of the samples examined via monitoring the discoloration of the dye via optical absorption studies using UV-VIS spectrophotometer (JASCO V-570, Tokyo, Japan). The samples were also tested against Methylene blue dye, where the same procedure was followed using 10 ppm dye concentration

## 3. Results and Discussion

**Figure 1:**
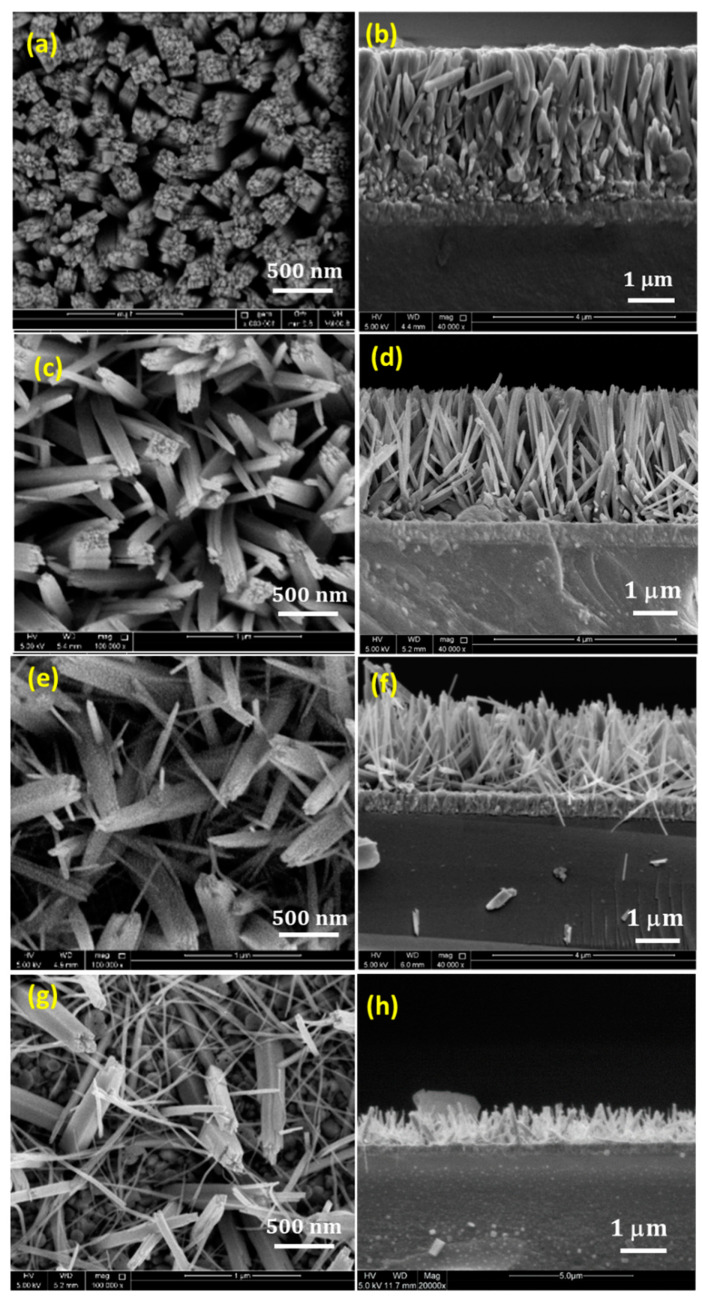
Top view and cross-sectional SEM images of TiO_2_ NRs with: (**a**,**b**) 0 M, (**c**,**d**) 0.05 M, (**e**,**f**) 0.1 M, (**g**,**h**) 0.15 M KBr.

The morphological characteristics of the TiO_2_ NRs grown with different KBr concentrations were examined by SEM. Figure 1 represents the top view and cross-sectional SEM images of the as-prepared TiO_2_ NRs samples with different KBr concentrations. Figure 1a,b represent the top view and the cross sectional images of the TiO_2_ NRs sample grown without any addition of KBr. It can be seen that the substrate is uniformly covered with densely aligned and ordered nanorods with uniform height. These nanorods exhibit tetragonal pillar geometrical shape, with square top surface, that is consistent with the growth of tetragonal crystal structure of TiO_2_ in hydrothermal synthesis. The nanorods exhibit an average diameter of 290 nm, 3.1 μm length, and very small separation between nanorods at the top and clustering of nanorods at the bottom. Such structure may prevent the light exposure and the electrolyte penetration to the side surface of the nanorods. Thus, enhancing the free space between the NRs is necessary to maximize the surface side exposure. By the addition of KBr salt, it can be noticed that the length, diameter, as well as the alignment of the grown nanorods are affected. Figure 1c,d show the effect of adding 0.05 M KBr, where the length of the nanorods slightly decreases to 2.9 μm, and the average diameter of the nanorods decreases to 123 nm, providing more spaces between the nanorods. The formation of these interstitial spaces between the adjacent nanorods, results in enlarging the inner surface area of the NRs with more side exposure. In addition, increasing the internal surface area between the NRs is believed to facilitate the coupling with other materials for the formation of heterostructures. By further increasing the concentration of the KBr, the diameter and the length of the nanorods decrease significantly. Adding 0.1 M KBr causes the diameter and the length of the NRs to drop to an average of 65 nm and 2 μm, respectively, as shown in Figure 1e,f. This reduction in the diameter causes the bending of the nanorods [15], where they start to appear randomly aligned instead of growing normal to the substrate surface. When the growth was conducted by adding 0.15 M KBr, the TiO_2_ morphology tends to appear like nanofibers where the length dramatically reduced into 0.4 μm with totally random alignment, as shown in Figure 1g,h. It can be seen that the crystal morphology of the NRs can be tuned by introducing the KBr molecules into the solution, which preferentially could be absorbed to different crystal faces, modifying the surface energy and suppressing the growth along specific direction. The addition of KBr plays an important role in inhibiting the lateral growth of the NRs and allowing the growth in the axial direction. This can be seen clearly from the reduction in the diameter, as well as the formation of free spaces between the adjacent NRs. The role of KBr in controlling the diameter and the length of the grown nanorods could have some possible explanations. The presence of KBr salt would increase the ionic strength of the growth solution significantly [27,28]. A high ionic strength solution is believed to reduce the overall growth rate, which will favor the formation of small crystals. This explains the observed reduction in the length and the diameter. Moreover, the presence of the salt ions during the growth will contribute to the lateral growth inhibition by being adsorbed on the lateral plane of the NRs. Thus, they can act as a diffusion barrier retarding the precursors to be diffused, and this explains the significant reduction in the diameter of the NRs [15]. Over all, the addition of KBr has a considerable control over the diameter, alignment and the inner surface area of the prepared NRs.

**Figure 2:**
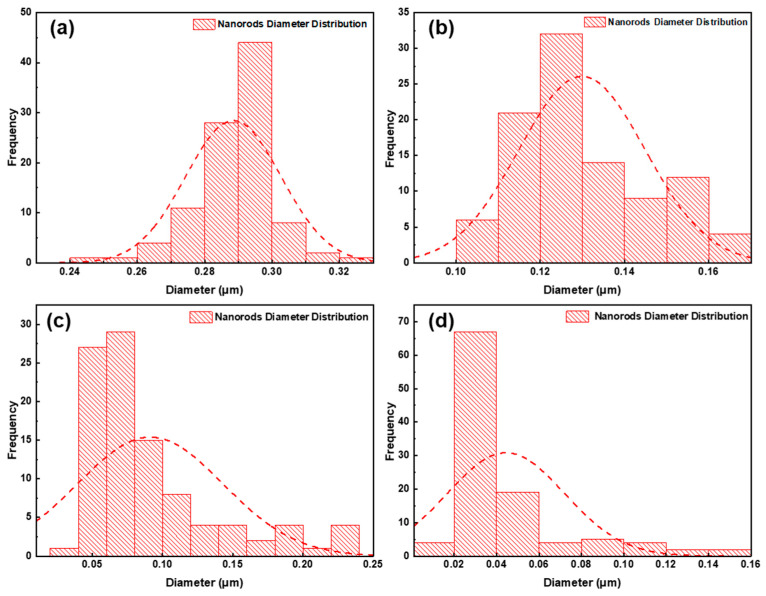
Distribution of TiO_2_ nanorods diameter in (**a**) 0 M KBr, (**b**) 0.05 M, (**c**) 0.1, (**d**) 0.15 M samples.

Size statistics of the diameter and distribution of the TiO_2_ nanorods for the samples with different KBr concentrations are presented in Figure 2. It can be seen that as the concentration of KBr increased, the average diameter of the TiO_2_ nanorods is decreased, and the range of size distribution increase.

A proposed mechanism for the influence of KBr in the formation of TiO_2_ NRs is given in Equations (1)–(4). Equations (1) and (2) represent the formation of TiO_2_ NRs with the absence of KBr, where the only ion species present in the solution is chloride ion (Equation (2)). By adding KBr, the growth solution will contain more ions species (Cl^−^, Br^−^, and K^+^), where they will increase the ionic strength of the solution, and act as a diffusion barrier (Equation (4)).

(1) $$HCl_{\left(l\right)}+H_{2}O_{\left(l\right)}⇌H_{3}O^{+}+Cl^{-}$$

(2) $$Ti{\left(OCH_{2}CH_{2}CH_{2}CH_{3}\right)}_{4}+2H_{3}O^{+}+2Cl^{-}\overset{heat}{\to }TiO_{2}+4{\left(CH\right)}_{4}CH_{3}OH+2Cl^{-}+2H^{+}$$

(3) $$HCl_{\left(l\right)}+H_{2}O_{\left(l\right)}+KBr⇌H_{3}O^{+}+Cl^{-}+K^{+}+Br^{-}$$

(4) $$Ti{\left(OCH_{2}CH_{2}CH_{2}CH_{3}\right)}_{4}+2H_{3}O^{+}+2Cl^{-}+K^{+}+Br^{-}\overset{heat}{\to }TiO_{2}+4{\left(CH\right)}_{4}CH_{3}OH+2Cl^{-}+K^{+}+Br^{-}+2H^{+}$$

**Figure 3:**
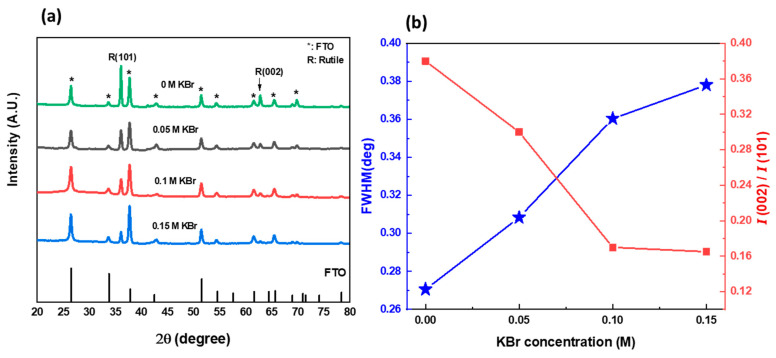
(**a**) XRD patterns of TiO_2_ nanorods with different KBr concentrations (0 M; 0.05 M; 0.1 M; and 0.15 M). (**b**) Variation of the XRD FWHM and integral intensity of (002)/(101) plane of TiO_2_ nanorods with KBr concentration.

The structural formation of the TiO_2_ NRs via hydrothermal reaction at different KBr concentrations was characterized using X-ray diffraction. Figure 3a shows the XRD patterns of TiO_2_ NRs samples with 0 M, 0.05 M, 0.1 M, and 0.15 M KBr concentrations. It can be observed that all the samples showed the phase formation of the crystalline structure of tetragonal P4_2_/mnm Rutile crystal with the lattice constants 4.594 Å and 2.960 Å for a and c, respectively. However, the samples showed different degree of crystallinity. The diffraction patterns for all samples show two major peaks at 36° and 62.7°, corresponding to crystal planes of (101) and (002), respectively [3]. The intensity ratio of (002) peak over (101) peak can give information about the degree of alignment of the grown TiO_2_ nanorods [29,30]. Figure 3b plots the integrated intensity ratio (002)/(101) and XRD full width at half maximum of (101) plane of TiO_2_ nanorods as a function of KBr concentration. It can be seen that 0 M KBr sample exhibits the highest *I*(002)/*I*(101) ratio, which indicates that the nanorods are highly oriented with respect to the substrate surface, suggesting the preferential growth of the film in the [001] directions, with the growth axial parallel to the substrate normal. As the concentration of the KBr increases, the (002)/(101) ratio decreases, suggesting that the alignment of the grown nanorods starts to decrease along the [001] direction. These conclusions are supported by the SEM results in Figure 1. Figure 3b also shows clearly that the FWHM of the (101) plane increases almost linearly with increasing KBr concentration and the intensity is strongly reduced. This can be explained by the etching role of KBr, whereas the concentration of KBr increases, the diameter of the nanorods decreases. Thus, this decrease in the crystal dimensions leads to broader diffraction peak, with bigger FWHM. Regardless of the overall decrease in the intensity, all samples exhibited identical diffraction peaks’ positions, which demonstrates that the KBr addition does not damage the crystal structure of the prepared TiO_2_ NRs.

**Figure 4:**
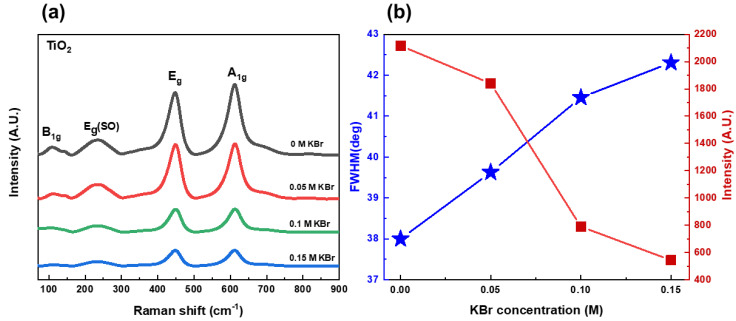
(**a**) Raman spectra of TiO_2_ nanorods grown hydrothermally with different KBr concentrations (0 M; 0.05 M; 0.1 M; and 0.15 M). (**b**) FWHM and intensity of Eg active mode peak as a function of KBr concentration.

To study the structural properties and the possible presence of other phases in the samples, Raman spectra were obtained, as shown in Figure 4a. Raman scattering in all samples confirms the formation of Rutile phase TiO_2_ with a space group of P4_2_/mnm. The Raman spectra exhibit four main peaks. Three of these peaks are Raman active modes that appeared at 143 cm^−1^, 442 cm^−1^, and 607 cm^−1^, corresponding to B_1g_, E_g,_ and A_1g_ active modes. One more peak observed at 235 cm^−1^ denotes second-order scattering by disorder rutile lattice [31]. Figure 4b plots the FWHM and the intensity of the Eg active mode peak as a function of KBr concentration. The FWHM was evaluated using Origin software, by applying Gaussian fitting. It can be seen that the FWHM increases almost linearly with an increase in KBr concentration, and the intensity of the Eg active mode peak is reduced with KBr concentration. This agrees with XRD results, where the decrease in the nanorods diameter results in broader peaks.

From the above results, it can be concluded that the TiO_2_ NRs sample with 0.05 M KBr concentration exhibits a good inner separation between the TiO_2_ NRs with reasonable NRs length (~2.9 μm) and alignment, and crystalline quality. Thus, it is going to be used for further characterization and construction of MoS_2_/TiO_2_ NRs heterostructures.

**Figure 5:**
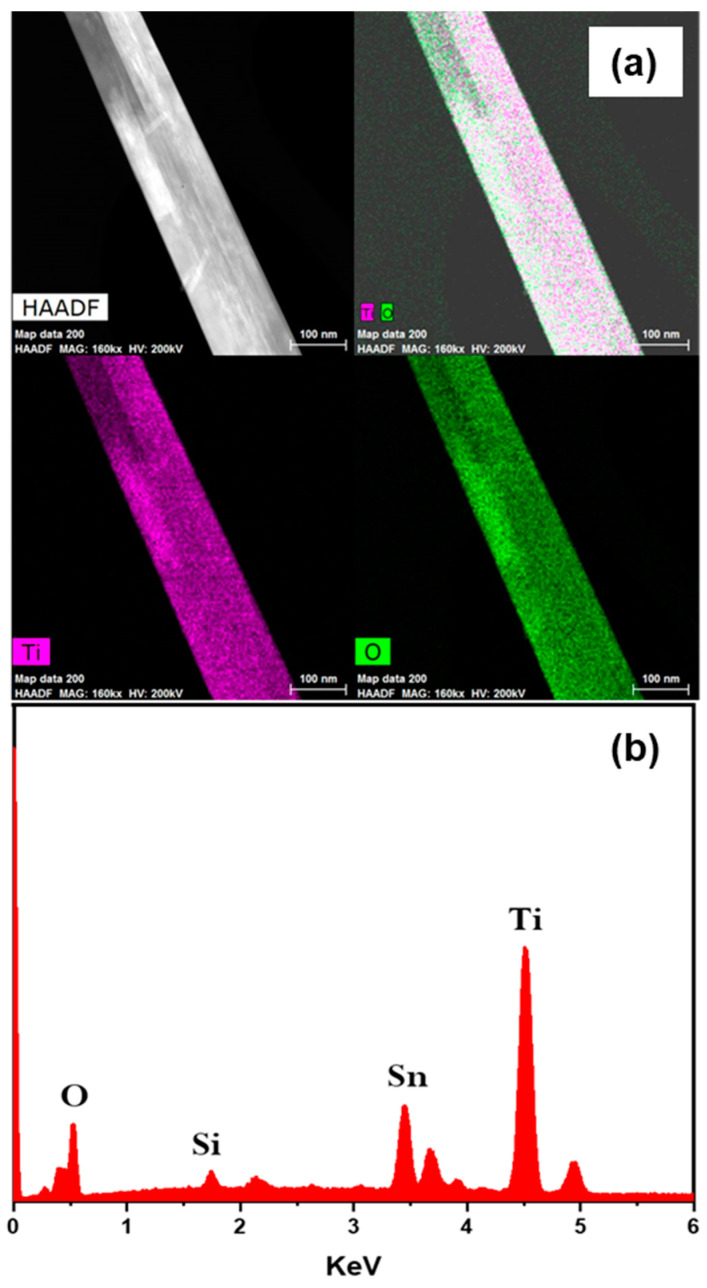
EDS chemical characterization of TiO_2_ nanorods grown with 0.05 M KBr (**a**) showing high-angle annular dark-field (HAADF) image and the corresponding Ti and O mappings in pink and green, respectively (**b**) EDS spectra.

To explore the microstructure of the grown TiO_2_ NRs, TEM analysis was carried out for the sample with 0.05 M KBr. X-ray energy dispersive spectroscopy (EDS) elemental mapping was performed to give better understanding about the chemical composition of the nanorods, as shown in Figure 5a. The mapping confirmed that the nanorods are composed of titanium (Ti), and oxygen (O) elements, in which the Ti and O elements are distributed throughout the whole rod, homogeneously. It is also confirmed that the rod has a diameter of around 120 nm, which agrees with the average diameter in the SEM analysis as shown in Figure 1c. Figure 5b shows the EDS spectra, where the main detected elements are Ti and O, with some presence of Sn coming from the FTO substrate.

**Figure 6:**
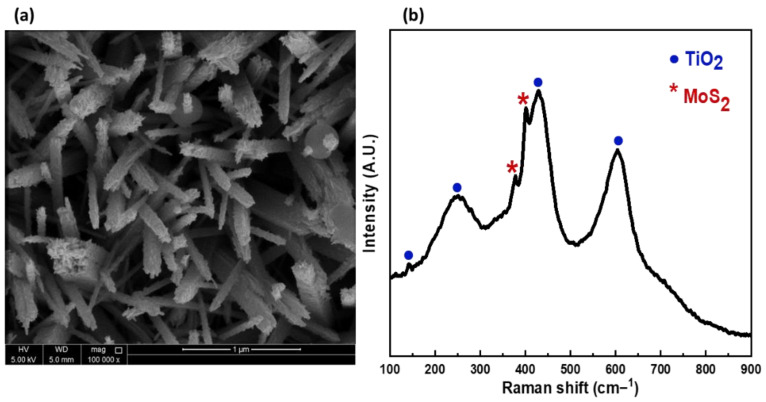
(**a**) SEM top view image, (**b**) Raman scattering spectrum of MoS_2_/TiO_2_ heterostructure.

As discussed previously, the sample with 0.05 M KBr exhibits aligned NRs with larger inner spacing between the NRs compared to the sample without KBr. It is believed that this enhanced spacing between the NRs will facilitate the construction of MoS_2_/TiO_2_ heterostructure. The hydrothermally grown TiO_2_ NRs on FTO was used as a substrate for the deposition of MoS_2_ vertical nanoflakes. Figure 6a shows the SEM top view image of TiO_2_ NRs with deposited MoS_2_ nanoflakes. It can be seen that plentiful of MoS_2_ vertical nanoflakes were grown on the surface of the as-prepared TiO_2_ NRs to confirm the formation of MoS_2_/TiO_2_ heterostructure. It also can be noticed that both top faces and side surfaces of the NRs were roughened by MoS_2_ nanoflakes, which could be in favor of incident light absorption for photoactivity. The worth noting point is that the influence of KBr in enhancing the free space between the adjacent NRs provide more inner surface area for the deposition of MoS_2_ vertical nanoflakes and for the incident light and electrolyte penetration. For further confirmation, Raman spectrum was obtained, as shown in Figure 6b. It can be seen that the spectrum exhibits two peaks at around ∼381.0 and ∼402.0 cm^−1^, which correspond to the E_2g_^1^ and A_1g_ vibrational modes of MoS_2_, respectively [32]. Moreover, it exhibits the three main peaks of the TiO_2_ Raman active modes at 150 cm^−1^, 442 cm^−1^, and 607 cm^−1^, corresponding to B_1g_, Eg_,_ and A_1g_, respectively. Subsequently, MoS_2_/TiO_2_ heterostructure sample exhibits Raman signatures for both TiO_2_ and MoS_2_, which indicates the successful growth of MoS_2_ vertical nanoflakes on the TiO_2_ NRs. The MoS_2_/TiO_2_ heterostructure was further characterized by XRD and EDS as shown in the supporting data. Figure S3 shows the XRD pattern of the MoS_2_/TiO_2_ heterostructure sample. It can be seen that diffraction peaks at 14.5°, and 28.6° are consistent with the MoS_2_ hexagonal phase, which can be attributed to the (002) and (004) planes. Moreover, the pattern shows two diffraction peaks at 36° and 62.7°, corresponding to TiO_2_ crystal planes of (101) and (002) respectively, revealing the phase formation of rutile crystal structure. Figure S4 shows the EDS spectra of the as-prepared MoS_2_/TiO_2_ heterostructure. It can be seen that the main detected elements are Ti, O, Mo, and S, giving a strong indication of the successful synthesis of MoS_2_/TiO_2_ heterostructure.

## 4. Photocatalytic Degradation of Rhodamine B

The photocatalytic activity of as-prepared TiO_2_ NRs with different KBr concentrations, as well as the MoS_2_/TiO_2_ heterostructure sample, were evaluated towards the degradation of Rhodamine B dye under solar simulator irradiation. Figure S1 represents the absorption spectra of the Rhodamine B dye degradation using MoS_2_/TiO_2_ heterostructure sample as a photocatalyst. It is clear that the intensity of the Rhodamine B absorption band at 554 nm exhibits a reduction with increasing the degradation time, which indicates the photocatalytic activity of the sample. The concentration C of the dye solution was obtained via the standard curve method. To calculate the degradation efficiency of the used photocatalysts, following equation was used [33]:

(5) $$\%\;D=\frac{C_{0}-C}{C_{0}}\times 100$$

where *C*_0_ is the initial concentration and *C* is the concentration of the dye at a relative exposure time *t*.

The Langmuir–Hinshelwood model was used to calculate the reaction rates of Rhodamine B degradation by the pseudo-first-order kinetic equation [33]:

(6) $$\ln\left(\frac{C_{0}}{C}\right)=kt$$

where *k* is the reaction rate constant.

**Figure 7:**
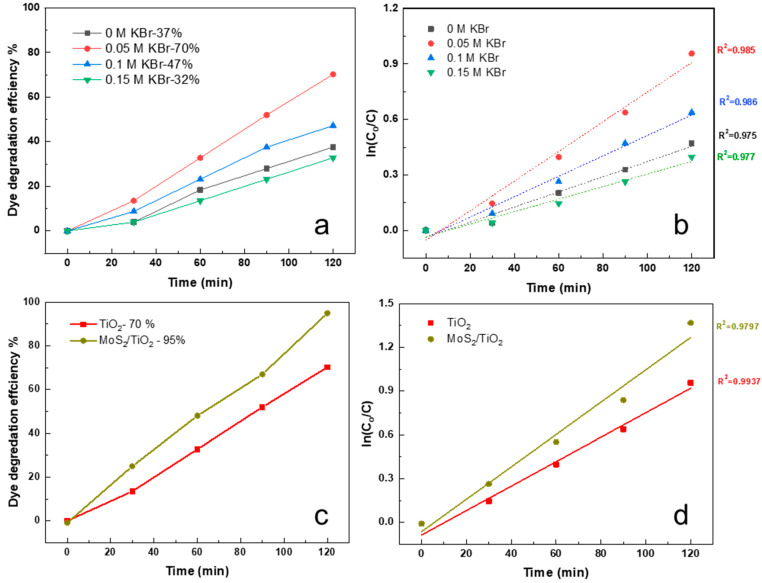
(**a**,**b**) dye degradation efficiency and kinetic plots of TiO_2_ NRs with different KBr concentrations, (**c**,**d**) dye degradation efficiency and kinetic plots of MoS_2_/TiO_2_ heterostructure and TiO_2_ NRs with 0.05 M KBr.

Figure 7a shows the degradation efficiency (%) versus time (min) graph of the as-prepared TiO_2_ NRs samples with different KBr concentrations. It can be observed that 0.05 M KBr sample exhibits the highest dye degradation efficiency with 70%. Further increase in the KBr concentration to 0.1 M drops the efficiency to 47%. However, it still exhibits higher activity than the sample with 0 M KBr concentration which gives 37%. This variation in the activity is related to the effect of KBr on the morphology of the samples. As discussed in Figure 1, 0 M KBr sample exhibits high density NRs with very small spacing between the NRs. In this case, there is no exposure to the side surface of the NRs, where only the top surfaces are exposed, resulting in minimizing the inner surface area. This may explain the weak photocatalytic activity with less exposed surface area. The addition of 0.05 M of KBr causes a reduction in the diameter of the nanorods, providing more gap spaces between the adjacent nanorods, and side surface exposure to the incoming radiation. By increasing the concentration to 0.1 M, further reduction in the diameter was observed with further exposure to the radiation. This may explain the enhancement achieved when compared to the 0 M concentration, where it has larger inner surface area exposed to the light. However, the significant reduction in the diameter caused the bending of the NRs and the random alignment, which resulted in the escape of the radiation, thus weakening their photocatalytic activity [34]. Further increase in the concentration of the KBr to 0.15 M gives the lowest activity with 32% degradation efficiency. This can be directed to the formation of very small nanowires; with a significant decrease in their length, diameter, and alignment. The first order kinetic expression showed in equation 6 was used to fit the photodegradation reaction as shown in Figure 7b. For all samples, a linear relationship can be observed between ln(*C*_0_/*C*) and the irradiation time t that following the first-order kinetic reaction. The rate constant k for 0 M, 0.05 M, 0.1 M, and 0.15 M was calculated as 0.0045, 0.0106, 0.0059, and 0.0037 min^−1^, respectively, as shown in Table 1.

The photocatalytic behavior of the MoS_2_/TiO_2_ heterostructure was also tested to degrade Rhodamine B dye. Figure 7c compares the degradation efficiency between MoS_2_/TiO_2_ heterostructure sample and the TiO_2_ NRs with 0.05 M KBr sample. The degradation efficiency achieved by the heterostructure sample is around 1.36 times higher than that of the TiO_2_ NRs sample, with a higher rate constant as shown in Figure 7d. The as prepared heterostructure was also tested against methylene blue dye; however, it does not show a promising activity, where it exhibits only 20% degradation as shown in Figure S2.

**Figure 8:**
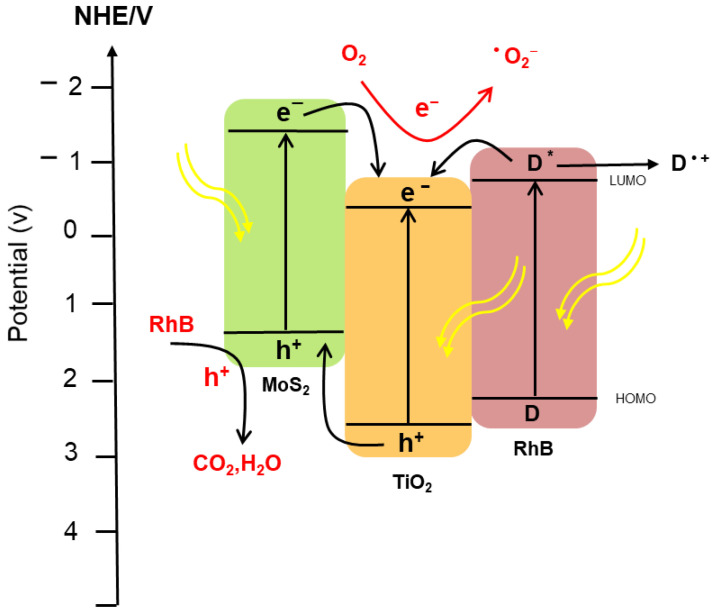
Proposed mechanism of photocatalytic degradation of RhB dye over MoS_2_/TiO_2_ catalyst.

The possible mechanism for the photocatalytic degradation of RhB by MoS_2_/TiO_2_ heterostructure is illustrated in Figure 8. The band energy of TiO_2_ and MoS_2_ were found to be 3.16 eV and 1.86 eV, respectively [35,36]. According to the previous values, the mechanism of photocatalytic degradation was constructed. When the heterostructure sample is irradiated with a solar simulator, electronic excitation will occur in both materials, with less light harvesting in TiO_2_. The electrons that get excited to the CB of MoS_2_ will be transferred to the CB of TiO_2_. This transfer will occur because the CB edge potential of MoS_2_ is more negative than the CB value of TiO_2_. RhB dye will also absorb some photons where the electrons will get excited from the HOMO level to the LUMO level. The LUMO level of RhB is also more negative than the CB of TiO_2_; thus, electrons will be injected from the RhB LUMO level to TiO_2_ CB. Therefore, TiO_2_ CB will act as a sink to trap the electrons from MoS_2_, RhB as well as the electrons excited from the material itself. These electrons will participate in the reduction of O_2_ to form ^.^O_2_^−^, as the CB potential of TiO_2_ is more negative than the standard potential of O_2_/^.^O_2_^−^ (−0.33 V/NHE). These generated superoxide radicals will work on oxidizing the dye and the cationic radicals into H_2_O, CO_2,_ and other mineral salts. Another charge transfer is proposed to happen for the holes presented in the VB of TiO_2_ into the VB of MoS_2_, since it is more positive. These injected holes from TiO_2_ along with holes lifted from the electrons’ excitation in MoS_2_ will participate in the oxidation of the RhB molecules into degraded products. Since the VB potential of MoS_2_ is less positive than the standard redox potential of ^.^OH/OH^−^, the holes accumulating there will not be able to oxidize OH^−^ to form ^.^OH [37,38].

## 5. Conclusions

In summary, effects of adding KBr salt during the hydrothermal growth of TiO_2_ nanorods were investigated. It was found that tuning the KBr concentration controls the alignment and the morphology parameters such as length, diameter, spacing, and density of TiO_2_ nanorods. Which will lead to the increase of specific surface area of the TiO_2_ nanorods, facilitating the coupling with other materials for the formation of heterostructures. The crystallinity of the TiO_2_ nanorods was found to decrease almost linearly with the increase in KBr concentration in the growth solution. The results have shown that the optimal KBr concentration for coupling of TiO_2_ nanorods with MoS_2_ nanoflakes and for achieving high photocatalytic degradation efficiency of RhB dye is 0.05 M. This can be directed to the enlargement in the inner surface area, which provides more exposure to side surface. The ability of tuning nanorods morphology opens up the possibility of engineering complex structures such as heterostructures with enhanced performance in different applications such as catalysis and batteries.

## Figures and Tables

**Table 1:** Rate constant values of TiO_2_ samples with different KBr concentration.

Sample	Rate Constant K (min^−1^)
**0 M KBr—TiO_2_**	**0.0045**
**0.05 M KBr—TiO_2_**	**0.0106**
**0.1 M KBr—TiO_2_**	**0.0059**
**0.15 M KBr—TiO_2_**	**0.0037**

## Data Availability

Data supporting the findings of this study are available by reasonable request to: taltahtamouni@qu.edu.qa.

## Supplementary Materials

The following supporting information can be downloaded at: https://www.mdpi.com/article/10.3390/nano12172904/s1, Figure S1: Absorption spectra of RhB dye at different degradation time using MoS_2_/TiO_2_ heterostructure; Figure S2: Dye degradation efficiency of MoS_2_/TiO_2_ heterostructure against Methylene blue dye; Figure S3: XRD pattern of MoS2/TiO_2_ heterostructure; Figure S4: EDS chemical spectrum of MoS_2_/TiO_2_ heterostructure.
