# Supplementary material for: Morphology Control of TiO2 Nanorods Using KBr Salt for Enhancing the Photocatalytic Activity of TiO2 and MoS2/TiO2 Heterostructures
Source: Nanomaterials (Basel). 2022 Aug 24;12(17):2904. doi: 10.3390/nano12172904 (PMC9457778; doi:10.3390/nano12172904)
Supplement: Supplementary file 1 [file nanomaterials-12-02904-s001.zip › nanomaterials-1671383-supplementary.pdf]

Supporting Information for

Morphology control of TiO_2 nanorods using KBr salt for enhancing the photocatalytic activity of TiO_2 and MoS_2/TiO_2 heterostructures

Zeineb Thiehmed[1], Talal. M. Al tahtamouni[1,*]

[1]Materials Science & Technology Program, College of Arts & Sciences, Qatar University, Doha 2713, Qatar

* Corresponding author. Email: taltahtamouni@qu.edu.qa

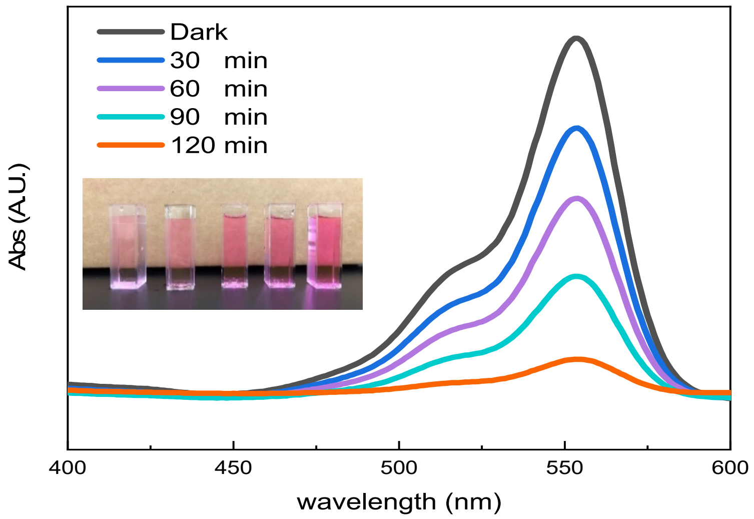

Figure S1: Absorption spectra of RhB dye at different degradation time using MoS_2/TiO_2 heterostructure.

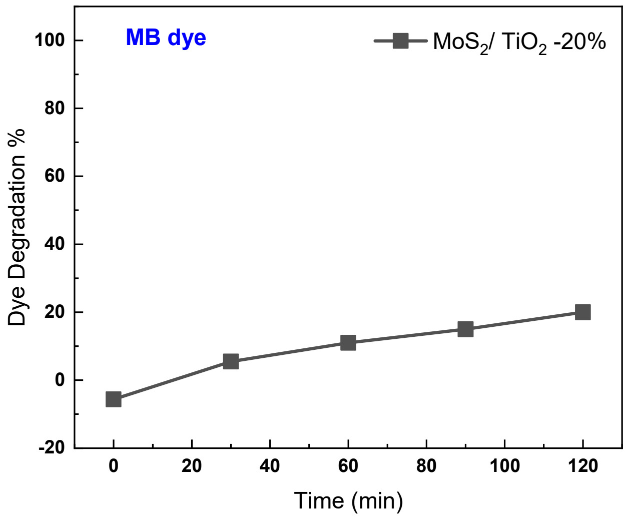

Figure S2: Dye degradation efficiency of MoS_2/TiO_2 heterostructure against Methylene blue dye

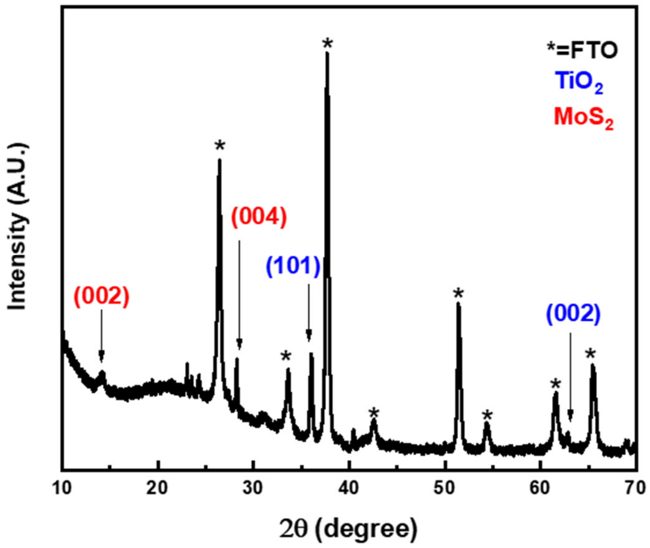

Figure S3: XRD pattern of MoS_2/TiO_2 heterostructure

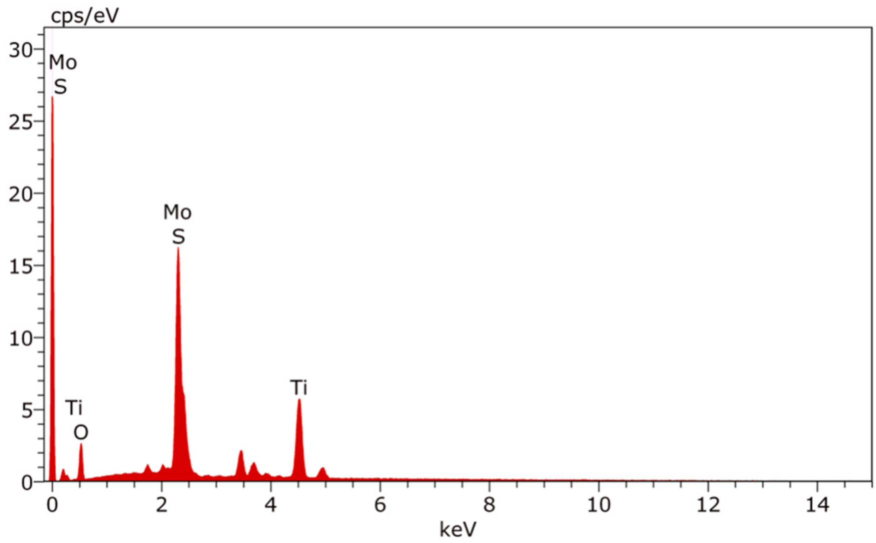

Figure S4: EDS chemical spectrum of MoS_2/TiO_2 heterostructure
